# Comparative study between I-gel, a new supraglottic airway device, and classical laryngeal mask airway in anesthetized spontaneously ventilated patients

**DOI:** 10.4103/1658-354X.71250

**Published:** 2010

**Authors:** Amr M. Helmy, Hossam M. Atef, Ezzat M. El-Taher, Ahmed Mosaad Henidak

**Affiliations:** *Department of Anesthesia and Intensive Care, Faculty of Medicine, Suez Canal University, Ismailia, Egypt*

**Keywords:** *Classical laryngeal mask airway*, *I-gel*, *supraglottic airway devices*

## Abstract

**Objective:**

To compare two different supraglottic airway devices, the laryngeal mask airway (LMA) and the I-gel, regarding easiness of insertion of the device, leak pressure, gastric insufflation, end tidal CO_2_, oxygen saturation, hemodynamic and postoperative complications in anesthetized, spontaneously ventilated adult patients performing different non-emergency surgical procedures.

**Materials and Methods:**

The study was carried out as a prospective, randomized, clinical trial among 80 patients who underwent different surgical procedures under general anesthesia with spontaneous ventilation in supine position. They were equally randomized into two groups: I-gel and LMA groups. Both the devices were compared with regard to heart rate, arterial BP, SPO_2_, end-tidal CO_2_, number and duration of insertion attempts, incidence of gastric insufflation, leak pressure and airway assessment after removal of the device.

**Results:**

No statistically significant difference was reported between both the groups, regarding heart rate, arterial BP, SPO_2_ and end-tidal CO_2_. The mean duration of insertion attempts was 15.6±4.9 seconds in the I-gel group, while it was 26.2±17.7 seconds in the LMA group. The difference between both the groups regarding duration of insertion attempts was statistically significant (*P*=0.0023*), while the number of insertion attempts was statistically insignificant between both the study groups (*P*>0.05). Leak pressure was (25.6±4.9 vs. 21.2±7.7 cm H_2_O) significantly higher among studied patients of the I-gel group (*P*=0.016*) and the incidence of gastric insufflation was significantly more with LMA group 9 (22.5%) vs. I-gel group (5%) (*P*=0.016).

**Conclusion:**

Both LMA and I-gel do not cause any significant alteration in the hemodynamic status of the patients, end tidal CO_2_, and SPO_2_. The postoperative complications were not significantly different except nusea and vomiting was statistically significant higher in LMA group (*P*=0.032). among both LMA and I-gel patients. Insertion of I-gel was significantly easier and more rapid than insertion of LMA. Leak pressure was significantly higher with I-gel than LMA and thus incidence of gastric insufflation was significantly lower with I-gel.

## INTRODUCTION

The major responsibility of the anesthesiologist is to provide adequate ventilation to the patient. The most vital element in providing functional respiration is the airway. Management of the airway has come a long way since the development of endotracheal intubation by Macewen in 1880 to the present day usage of sophisticated devices.[1] The tracheal intubation is the gold standard method for maintaining a patent airway during anesthesia.[2] However, this maneuver requires skill and continuous training and practice and usually requires direct laryngoscopy, which may cause laryngopharyngeal lesions.[3]

Laryngoscopy and endotracheal intubation produce reflex sympathetic stimulation and are associated with raised levels of plasma catecholamines, hypertension, tachycardia, myocardial ischemia, depression of myocardial contractility, ventricular arrhythmias and intracranial hypertension.[4] The wide variety of airway devices available today may broadly be classified as intraglottic and extraglottic airway devices, which are employed to protect the airway in both elective as well as emergency situations.[5]

The laryngeal mask airway (LMA; Laryngeal Mask Company, Henley-on-Thames, UK) has been well established for more than a decade and is often used when endotracheal intubation is not necessarily required.[6] Nevertheless, simple handling of the LMA is limited by the potential risk of aspiration[7] (because fiberoptic studies have found ~6–9% visualization of the esophagus via the LMA)[8,9] or low pulmonary compliance [(e.g. obesity) requiring peak inspiratory pressures greater than 20 cm H_2_O].[10]

I-gel is the single use supraglottic airway from intersurgical, UK (Intersurgical Ltd, Wokingham, Berkshire, UK) with an anatomically designed mask made of a gel like thermoplastic elastomer. It has features designed to separate the gastrointestinal and respiratory tracts and allow a gastric tube to be passed into the stomach.[4] The tensile properties of the I-gel bowl, along with its shape and the ridge at its proximal end, contribute to the stability of the device upon insertion. Upon sliding beneath the pharyngo-epiglottic folds, it becomes narrower and longer, creating an outward force against the tissues. The ridge at the proximal bowl catches the base of the tongue, also keeping the device from moving upward out of position (and the tip from moving out of the upper esophagus).[11]

The main aim of this study was to compare the I-gel with the LMA in terms of the success of insertion of the device, gas leak pressure, the incidence of gastric insufflations and postoperative device related complications.

## MATERIALS AND METHODS

### Subjects

This study was carried out as a prospective, randomized, clinical trial. After getting approval from our ethics committee, 80 patients aged 21–60 years, of both sexes, who underwent different surgical procedures under general anesthesia with spontaneous ventilation in supine position for not more than 2 hours in routine surgical theaters in Suez Canal University Hospital in Ismailia city, were enrolled based on certain inclusion and exclusion criteria. Inclusion criteria included the following: (i) patients of ASA I or ASA II and (ii) patients whose body mass index (BMI) was 20–25 kg/m^2^. Patients with reported history of hypersensitivity for one or more of the medications and latex, patients having any abnormality of the neck, upper respiratory tract, patients with history of obstructive sleep apnea or patients who underwent thoracic, abdominal and neurosurgery operations were excluded. The patients were equally randomized into two groups: group 1 (I-gel group) and group 2 (LMA group).

### Methods

#### Preoperative assessment and medication

Complete medical history and physical examination were done for all patients, including assessment of vital signs and airway assessment. After arrival in the pre-anesthetic area, the patients were given 2 mg midazolam intravenously (IV) as premedication, and then 10 mg Metoclopramide IV was also given 3 minutes before induction of anesthesia Preoxygenation for 3 minutes, and anesthesia was induced with fentanyl 1mic/kg, and propofol 2 mg/kg.

#### Device insertion

After an adequate depth of anesthesia had been achieved, each device was inserted by the same senior anesthetist (A H). In group 1, the classical LMA was inserted according to the manufacturer’s instruction manual. A size 3 and 4 mask was used in females and a size 4 and 5 mask in males. The LMA cuff was inflated with 20 ml; 30 ml; 40 ml of air for size 3; 4; 5 respectively as recommended by the manufacture.[12]

For patients of group 2, the I-gel size #3, 4 or 5 was inserted according to the manufacturer’s instructions.[13]

In both the groups, if it was not possible to ventilate the lungs, the following airway maneuvers were allowed: chin lift, jaw thrust, head extension, or flexion on the neck. In the case of I-gel, the position was also allowed to be adjusted by gently pushing or pulling the device. After any maneuver, adequacy of ventilation was re-assessed. This maneuver was used with one patient in LMA group. If it was not possible to insert the device or ventilate through it, two more attempts at insertion were allowed. If placements had failed after three attempts, the case was abandoned and the airway maintained through other airway device as suitable and this case was considered as a failed attempt.

#### Maintenance of anesthesia

After securing the device, spontaneous ventilation in oxygen, air and inhalational agent was started. Ventilation was judged to be optimal if the following four tests will be passed: (i) adequate chest movement; (ii) stable oxygenation not less than 95%; (iii) “square wave” capnography and (iv) normal range end tidal CO_2_.

#### Removal of the device

At the end of the operation, anesthetic agents were discontinued, allowing smooth recovery of consciousness. The device was removed after the patient regained consciousness spontaneously and responded to verbal command to open the mouth. Dysphagia, dysphonia, nausea, vomiting and trauma of mouth, tooth or pharynx, and sore throat were recorded and reassessed within 24 hours.

#### Parameters measured

Monitoring equipments (Datex-Ohmeda™) were attached to the patient including 3 leads ECG and non-invasive blood pressure pulse oximetry and manometer was connected to the inspiratory limb of the breathing system to measure the airway pressure. The following parameters were measured.

1. Heart rate, non-invasive blood pressure, end-tidal CO_2_ tension and oxygen saturation (SpO_2_)
2. ‘The leak pressure by closing the expiratory valve of the circle system at a fixed low gas flow (3L/min), observing the air-way pressure at which equilibrium was reached. At this point, gas leakage was heard at the mouth, at the epigastrium (epigastric auscultation) or coming out the drainage tube (I-gel group). manometric stability test is one of the most reliable test.[14]
3. Number of insertion attempts and each attempt duration (time from picking up the device until attaching it to the breathing system in minutes).
4. Incidence of airway complications caused by supraglottic devices
  - reporting of post-extubation cough, breath holding or laryngeal spasm
  - observing presence of blood on the I-gel or LMA, and
  - lip and dental injury

Each patient was questioned to determine the following complications (in recovery room and 24 hours postoperatively): sore throat (constant pain, independent of swallowing), dysphagia (difficulty or pain with swallowing), sore jaw, dysphonia (difficulty or pain with speaking), numbness of the tongue or the oropharynx, blocked or painful ears, reduced hearing, or neck pain. Primary outcome measures: number and duration of insertion attempts, sealing pressure and peak airway pressure. Secondary outcome measures: postoperative airway complications. Power analysis was based on duration of insertion attempts (sec.) with Standard deviation (s) 15.62 and Variance (s_2_) 243.9. considering alpha (z_α_) error (*P*=0.05; therefore, 95% confidence desired (two-tailed test); z_α_=1.96) and beta (z) error (20% beta error, therefore, 80% power desired (one-tailed test); z_β_=0.84). Difference to be detected (d) 10 sec. Or larger difference between mean duration of the experimental group and control group 39 patients were required in each group.

### Statistical analysis

The Data was collected and entered into the personal computer. Statistical analysis was done using Statistical Package for Social Sciences (SPSS/version 17) software. A comparison of the overall abilities of the two techniques to accurately classify the patients was performed by a Z test to compare two portions. Arthematic mean, standard deviation, number and percent was calculated for each parameter. For categorized parameters chi-square test was used, Fisher exact test was used for data less than 5 in each cell, while for numerical data t-test was used to compare two groups. The level of significant was 0.05.[15]

## RESULTS

Analysis of the demographic characteristics of our patients under study has revealed that there was no statistically significant difference when comparing the mean age between the two groups (*P*>0.05). The same was found regarding the distribution of sex, as no statistically significant difference was found when comparing the two groups. Most of the patients in the groups of the study were found to be males (60 and 70% in I-gel and LMA groups, respectively). There was no statistically significant difference found in BMI between the two groups of the study (*P*>0.05) [Table 1].

**Table 1:** Personal characteristics of the patients under study

Characteristic	I-gel group (*n*=40)	LMA group (*n*=40)
Age	38.29±12.4	41.62±13.4
Gender		
Male	24 (60)	28 (70)
Female	16 (40)	12 (30)
BMI (kg/m^2^)	23.13±2.15	22.91±4.03

Data are presented as mean±SD or numbers (percentages); NS: no statistically significant difference (*P*>0.05), Figures in parentheses are in percentage

No statistically significant difference was found between both groups of the study, regarding each of systolic BP, diastolic BP, heart rate, SPO_2_ (%) and end-tidal CO_2_ throughout the whole duration of the surgery.

Table 2 shows that insertion and ventilation was possible at the first attempt in 90% of patients in the I-gel group and in 80% in LMA group. In 5% of the patients in LMA group, intubation and ventilation was possible after the third attempt. The mean duration of insertion attempts was 15.62±4.9 seconds in I-gel group, while it was 26.2± 17.7 seconds in LMA group. The difference between both groups regarding duration of insertion attempts was statistically significant (*P*=0.0023), while the number of insertion attempts was statistically insignificant between both the study groups (*P*>0.05). Leak pressure was significantly higher among patients of the I-gel group (25.62±4.9 versus 21.2±7.7 cm H_2_O in LMA group; *P*<0.016). The incidence of gastric insufflation was significantly more with LMA (22.5% versus 5% in I-gel group; *P*<0.016).

**Table 2:** Comparison between I-gel and LMA groups with respect to different parameters

Parameter	I-gel group (n=40)	LMA group (n=40)	*P* value
Number of insertion attempts			
One attempt	36 (90)	32 (80)	0.45 (NS)
Two attempts	3 (7.5)	6 (15)	
Three attempts	1 (2.5)	2 (5)	
Duration of insertion attempts (seconds)	15.62±4.9	26.2±17.7	0.0023*
Leak pressure (cm H_2_O)	25.62±4.9	21.2±7.7	0.016*
Incidence of gastric insufflation	2 (5)	9 (22.5)	0.016*

Data are presented as mean±SD or numbers (percentages); NS: no statistically significant difference (*P*>0.05);

* Statistically significant difference (*P*<0.05),

Figures in parentheses are in percentage

No statistically significant difference was found between both I-gel and LMA groups with regard to the assessment of patients after removal of the airway device [Table 3].

**Table 3:** Assessment of patients after device removal among the patients in both groups of the study

	I-gel group (n=40) (%)	LMA group (n=40) (%)	*P* value
Presence of blood on airway device	2 (5)	4 (10)	0.46 (NS)
Lip or dental injury	1 (2.5)	2 (5)	0.69 (NS)
Post removal cough	2 (5)	6 (15)	0.6 (NS)
Laryngeal spasm	4 (10)	4 (10)	1 (NS)
Sore throat			
Mild	12 (32.5)	10 (25)	0.34 (NS)
Moderate	3 (7.5)	5 (12.5)	
Dysphagia, dysphonia	2 (5)	2 (5)	1 (NS)
Postoperative nausea or vomiting	2 (5)	8 (20)	0.032*
Arrhythmia	2 (5)	3 (7.5)	0.69 (NS)
Pain on swallowing			
Mild	21 (52.5)	26 (65)	0.47 (NS)
Moderate	4 (10)	8 (20)	
Ear pain	3 (7.5)	5 (12.5)	0.46 (NS)
Hearing change	1 (2.5)	2 (5)	0.69 (NS)

NS: no statistically significant difference (*P*>0.05)

Success rate of gastric tube insertion was estimated to be 95%. Failed insertion was reported only among two patients (5%) [Table 4].

**Table 4:** Success rate of gastric tube insertion among the patients of I-gel group

		Number	Percent
Gastric tube insertion	Success	38	95
	Failure	2	5
Total		40	100

## DISCUSSION

The I-gel is a new supraglottic device, without an inflatable cuff, designed for use during anaesthesia.[11] It is a latex free, disposable device, made of a medical grade thermoplastic elastomer. I-gel is anatomically preformed to mirror the perilaryngeal structures. The device contains an epiglottis blocker, which helps to prevent epiglottis from downfolding or obstructing laryngeal inlet. The soft non-inflatable cuff seals anatomically against perilaryngeal structures. Furthermore, the I-gel has a gastric channel allowing venting of the air and gastric contents or insertion of gastric tube.[16]

It has features designed to separate the gastrointestinal and respiratory tracts and allow a gastric tube to be passed into the stomach. Early reports have postulated its use as a potential airway for use in resuscitation.[17] Many studies compared LMA with I-gel.[18–20]

Regarding the hemodynamic stability and effect of each of the supraglottic devices, no statistically significant difference was reported when comparing heart rate, systolic and diastolic arterial blood pressure throughout the surgery. Jindal *et al*.[21] reported hemodynamic stability with both LMA and I-gel devices, with no statistically significant difference between both devices, which is consistent with our findings.

Richez *et al*.[13] carried out one of the earliest studies to evaluate the I-gel. They found that insertion success rate was 97%. Insertion was easy and was performed at the first attempt in every patient. I-gel is easily and rapidly inserted, providing a reliable airway in over 90% of cases. Acott,[22] assessed the use of I-gel as an airway device during general anesthesia. In accordance with our results, they reported that a single insertion attempt was required in the majority of patients and all the insertion times recorded were less than 10 seconds. Similar results were obtained in study done by Gatward *et al*.,[23] who evaluated size 4 I-gel airway in 100 non-paralyzed patients and found that first insertion attempt was successful in 86% of patients, the second attempt in 11% of patients and the third attempt in 3% of patients.

Levitan and Kinkle[11] studied the positioning and mechanics of this new device in 65 non-embalmed cadavers with 73 endoscopies (eight had repeat insertion), 16 neck dissections, and 6 neck radiographs. A full view of the glottis (percentage of glottic opening score 100%) occurred in 44/73 insertions, whereas only 3/73 insertions had epiglottis-only views. Including the eight repeat insertions with a different size, a glottic opening score of >50% was obtained in all 65 cadavers. The mean percentage of glottic opening score for the 73 insertions was 82% (95% confidence interval 75–89%). In each of the neck dissections and radiographs, the bowl of the device covered the laryngeal inlet. They found that the I-gel effectively conformed to the perilaryngeal anatomy despite the lack of an inflatable cuff and it consistently achieved proper positioning for supraglottic ventilation.

The I-gel has potential advantages over other supraglottic airways for use by non-anesthetists during cardiopulmonary resuscitation. It has no cuff to inflate, making it simple to use. Its drain tube allows access to the gastrointestinal tract and it is designed to reduce the risk of gastric inflation and regurgitation. Simple airway maneuvers were required to assist in the placement but all devices were placed within two attempts.[24] These findings are consistent with our results.

One of the most important parameters to be compared between both supraglottic devices was postoperative complications. It was estimated that difference between LMA and I-gel regarding postoperative complications was not statistically significant except nausea and vomiting which was significantly higher in LMA due to high incidence of gastric insufflation. Consistent with our results, no major complications associated with I-gel have been described to date. Protection against aspiration is probably comparable with LMA family (but certainly not 100%). Minor complications reported include sore throat, temporary hoarseness, sore tongue, hyperesthesia of tongue.[13]

In the present study, only in two patients of the I-gel group, blood was on the device after removal. Acott,[22] did not report any case of blood on airway device (I-gel). In accordance with our results, he found that airway trauma during insertion of the I-gel was minimal.

Leak pressure was found to be significantly higher among patients of I-gel group than in LMA group (25.62 versus 21.2 cm H_2_O, respectively). This denotes that I-gel has better sealing pressure and that it fits well with the anatomy of supraglottic region. Acott[22] reported a leak pressure greater than 20 cm H_2_O in all patients.

Assessment of success rate of gastric tube insertion with I-gel was found to be 95%. This is consistent with what has been reported by Richez *et al*.,[13] as the gastric tube was inserted in 100% of cases. This helps in preventing gastric insufflation and decreasing air leak and thus decreasing postoperative nausea and vomiting.

A potential risk of the LMA is an incomplete mask seal, causing gastric insufflation or oropharyngeal air leakage.[25] Inconsistent with our findings; Schmidbauer and colleagues[26] concluded that both the ProSeal LMA and classical LMA provided better seal of the esophagus than the novel I-gel airway. Consistent with our results, Weiler *et al*.[6] had reported high incidence of gastric insufflation with the use of LMA.

There are some limitations of the present study. Firstly, we studied only low risk patients (ASA I and II) who had normal airways and were mostly not obese. Secondly, we did not compare performance with the likely competitors of the I-gel such as ProSeal LMA and laryngeal tube.

In conclusion, both LMA and I-gel do not cause any significant alteration in the hemodynamic status of the patients, end tidal CO_2_, and SPO_2_. The postoperative complications are not significantly different among both LMA and I-gel patients. Insertion of I-gel is significantly easier and more rapid than insertion of LMA. Leak pressure is significantly higher with I-gel than with LMA and thus incidence of gastric insufflation is significantly lower with I-gel.
